# Success of orthodontic treatment of linguoverted mandibular canine teeth using a direct inclined plane appliance

**DOI:** 10.3389/fvets.2023.1224391

**Published:** 2023-08-10

**Authors:** Leah Taylor, Lan Liu, Stephanie Goldschmidt

**Affiliations:** ^1^Texas Veterinary Dental Center, Stafford, TX, United States; ^2^School of Statistics, University of Minnesota, Twin Cities, Minneapolis, MN, United States; ^3^Department of Surgical and Radiologic Sciences, University of California, Davis, Davis, CA, United States

**Keywords:** linguoverted mandibular canine tooth, malocclusion, orthodontics, veterinary dentistry, base narrow, orthodontic retention

## Abstract

This study evaluated the success rate of orthodontic treatment of linguoverted mandibular canines in dogs using a directly applied inclined plane device. Medical records were retrospectively evaluated at 11 veterinary dental specialty hospitals from 1999 to 2021. Malocclusion classes included 41.7% in class 1, 47.2% in class 2, 6.9% in class 3, and 4.2% in class 4. The severity of linguoversion was mild in 7.6% of teeth, moderate in 33.9%, and severe in 58.5%. There was complete resolution of linguoversion in 71.2% of teeth, functional resolution in 25.4%, and failure in 3.4%. The median treatment time was 42 (11–174) days. Adjuvant orthodontic treatments were performed at the same time as the inclined plane in 45.7% of teeth, including active force orthodontics, extractions of non-strategic teeth, gingivectomy, and odontoplasty. While the inclined plane was in place, 31.4% of dogs required an anesthetized appliance adjustment, and at the time of appliance removal, complications occurred in 19.4% of dogs. Of the teeth that had initial resolution, 14.4% had rebound movement that required additional treatment. This study supports the idea that an acrylic inclined plane is a good treatment option for linguoverted mandibular canines, with a 96.6% success rate within a median of 6 weeks. Yet, orthodontic retention may be necessary in these cases to avoid the need for additional therapies.

## Introduction

Linguoverted mandibular canines (LMCs) appear to be a common abnormality in dogs. To the best of our knowledge, no epidemiologic studies evaluating the prevalence of LMCs in the canine population have been performed. Yet, in a retrospective study evaluating the distribution of malocclusions that presented to a veterinary dentistry specialty clinic over a 3-year period, 46.5% (98/198) of dogs had LMCs (1). An abnormal position of the canines can be associated with a dental (class 1) or skeletal (classes 2–4) malocclusion.^1^ Malocclusion may occur secondary to trauma, malpositioned tooth buds, toxins, tooth crowding, or genetics (2, 3). Regardless of the cause or specific class of malocclusion, untreated LMCs are associated with high morbidity and can result in both soft and hard tissue defects, including occlusal enamel trauma, oronasal fistula formation, and bone loss of the opposing maxillary teeth (4).

Mild cases of LMCs may be treated with ball therapy or gingivectomy/gingivoplasty with or without osteoplasty (5, 6). Treatment options for more severe LMC include orthodontic movement of the teeth to an atraumatic position, crown reduction with endodontic therapy (7, 8), or surgical extraction (9).

Orthodontic treatment and crown reduction are preferable to extraction as they do not compromise the bone integrity of the rostral mandible. The success rate for continued vitality with crown reduction with vital pulpotomy has been found to be 85%−100% (7, 8). Orthodontic treatment is the only option that allows for the maintenance of the complete mandibular canine tooth for aesthetic and functional purposes. Compared to crown reduction, orthodontic movement does not require ongoing radiographic evaluations (10).

Orthodontic treatment of LMCs can be performed with either crown extensions or an inclined plane (IP). Crown extensions utilize columns of acrylic or composite resin that encase the crown of the LMCs and extend dorsally and labially to engage the gingiva. On mouth closure, pressure is applied to the tooth to move it labially into an atraumatic position. Success rates for crown extensions are reported at 98.6% with a 25% complication rate (2). IP is an intermittent, passive force orthodontic device that is placed bilaterally on the maxillary arches to create a ramp to guide the LMCs into an atraumatic position. They can be fabricated and applied chairside or indirectly fabricated and applied at a second anesthetic procedure (11, 12). Chairside appliances are typically less expensive and can provide immediate treatment. Indirect appliances provide a custom fit and allow the use of alternative materials that may be better suited to prevent appliance damage in heavy chewers.

Previous case reports have shown success in the management of LMCs with this technique in both cats and dogs (12–16). Yet, no large-scale study exists that describes the success of this technique nor the time required for the successful movement of the LMCs to an atraumatic position. The aim of this study was to evaluate the success rate of directly applied IP for the treatment of LMC and determine factors that affect success.

## Methods and materials

Electronic medical records (EMR) were retrospectively reviewed from 11 veterinary dental specialist services from 1999 to 2021. Inclusion criteria included dogs with linguoversion of at least one mandibular canine tooth that was treated with a direct IP. Indirect IPs were excluded.

Clinical data collected from the medical records included breed, sex, weight, and age at the time of the appliance application. Labradoodles and Goldendoodles were recorded as Standard Poodles, while Labrador mixed-breed dogs were grouped with Labrador Retrievers. Additional information specific to the malocclusion, including the affected tooth/teeth, the presence of persistent deciduous teeth, and the class of malocclusion diagnosed, was recorded. The severity of the linguoversion was scored as previously described with minor modifications that included cases with insufficient space in the mandible to also be classified as severe (Table 1) (2).

**Table 1:** Grading criteria for severity of tooth displacement.

**Mild**	**The mandibular canine tooth cusp contacts the mucosa in the interdental space between the maxillary third incisor and canine**.
Moderate	The mandibular canine tooth cusp contacts the palatal tissue mesiopalatal to the maxillary canine tooth. There is no physical obstruction to orthodontic movement.
Severe	a. The mandibular canine tooth cusp contacts the palatal tissue mesiopalatal or palatal to the maxillary canine tooth. This requires more extensive movement for the affected tooth. b. The interdental space between the maxillary third incisor and canine is too narrow for the mandibular canine tooth. This is a physical obstruction for the tooth to reach an atraumatic position. c. Insufficient space to accommodate the mandibular canine tooth at the mandibular site. This is a physical obstruction for the tooth to reach an atraumatic position. d. Combination of severe changes.

Tooth displacement severity was graded on previously described criteria with modifications (2).

Procedural data collected from the EMR included tooth preparation for IP application, the material used to form the appliance, the teeth included, and the presence of a midline connection. The duration of treatment, defined as the time from when the appliance was placed to when it was removed, was recorded. Additional treatments and perioperative complications were recorded.

The outcome of treatment was scored based on a previously described grading scheme with modifications (2). Briefly, complete resolution was defined as the LMC resting labial to the gingival margin in an atraumatic position. Functional resolution was defined as the LMC cusp having ongoing tissue contact but causing no soft tissue (erythema, ulceration, and proliferative granulation tissue) or dental trauma (attrition). For both complete and functional resolution, no further treatment for the malocclusion was recommended. Incomplete resolution was defined as the LMCs requiring additional therapy due to ongoing trauma. The primary difference from the original grading scheme is that placement of a retention device did not automatically downgrade teeth to functional outcomes within our cohort (2).

If radiographs were performed at the time of removal, the findings from the radiology report were documented. Dental radiographs were not reviewed. Evaluation of the LMCs was provided, but information on additional teeth evaluated was not consistently available. Complications that occurred during removal, placement of a retention device, and follow-up data were recorded. In cases where a recheck physical examination was not documented in the EMR, follow-up was obtained through email or phone interviews with the referring veterinarian or owner.

### Statistical analysis

Data were collected separately for each LMC. A logistic regression model was used to analyze variables associated with treatment outcomes. The variables evaluated included the class of malocclusion, the age of the dog at the time of IP placement in days, the number of teeth incorporated in the appliance, and the total time the appliance was in place in days. For dogs that had different outcomes between the teeth, an additional analysis was performed based on the two outcomes. The worst-case scenario classified the entire dog based on the least successful outcome. The best-case scenario classified each dog by its most successful outcome. If a dog was re-treated, the age at the first treatment was used.

Spearman's rank correlation and a simple linear regression model were used to assess the relationship between the dog's age and treatment duration. To maintain independence between observations, age and treatment duration were averaged for dogs that were treated more than once. A one-way analysis of variance (ANOVA) and Tukey's honestly significant difference test were performed to determine the effect of the malocclusion type on the treatment duration.

Descriptive statistics were used to analyze the remaining data. Normally distributed continuous variables were reported as a mean and median. For the teeth that were retreated, the appliance construction data, complications, adjustments needed, additional treatment provided, duration of treatment, and outcome were included as a separate line in the data analysis.

All statistical analyses were performed in the R statistical software. The logistic regression models were fitted using the *lme4* library. The one-sample proportions test was conducted using the prop.test function. A *P*-value of <0.5 was considered statistically significant.

## Results

A review of the medical records identified 72 dogs with 118 LMC teeth treated with a direct IP. There were 113 teeth treated once and five teeth that were treated twice with the IP due to tooth rebound. There were 39 dog breeds, with Labrador Retrievers (15.3%; 11/72), German Shepherds (15.3%; 11/72), and Standard Poodles (13.9%; 10/72) being the most common. Breeds were not compared to hospital populations due to the multi-institutional nature of the study. Large-breed dogs were overrepresented with a median range weight of 23 (2–52.7) kg. There were 44.4% (32/72, 20 intact, 12 spayed) female and 55.6% (40/72, 30 intact, 10 neutered) male dogs. The median age (range) was 223 (159–964) days.

There was a history of persistent deciduous canine teeth on the affected side in 18% (13/72) of dogs and 36.4% (43/118) of teeth. Thirty-two were deciduous mandibular canine teeth, 20 of which were linguoverted. The remainder were maxillary canine teeth. Ninety-five percent (41/43) were extracted prior to IP placement, and the other two were naturally exfoliated. The timing of extractions was not consistently available in EMR.

**Figure 1:**
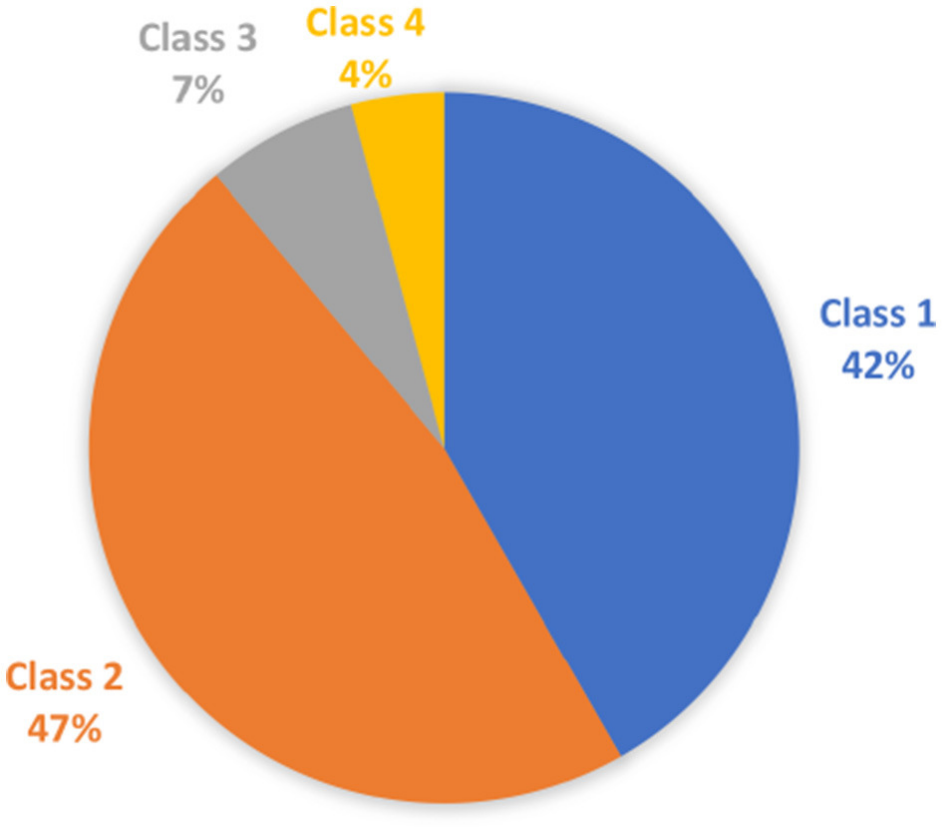
Classes of malocclusion in the study cohort. Pie chart depicting the classes of malocclusions treated with inclined plane in the study cohort.

Classes 1 and 2 malocclusions were most common (Figure 1). Notably, 7% (7.6%; 9/118) of teeth had mild displacement, 33.9% (40/118) had moderate displacement, and 58.5% (69/118) were classified as having severe displacement. Severe displacement was most common with class 2 malocclusions (Table 2).

**Table 2:** Distribution of the severity of displacement of each tooth by malocclusion class.

	**Class 1**	**Class 2**	**Class 3**	**Class 4**
Mild (%)	10.5	8.2	0	0
Moderate (%)	55.3	21.3	33.3	60
Severe (%)	34.2	70.5	66.7	40

Forty percent (40.3%; 29/72) of dogs had unilateral disease, and 59.7% (43/72) had bilateral disease. Two dogs initially diagnosed with bilateral disease had unilateral disease at the time of IP treatment due to prior orthodontic treatment. In one case, ball therapy resulted in the successful movement of one of the LMC. In another case, crown extension treatment successfully moved one tooth prior to IP placement. When there was unilateral disease, 71.4% (20/28) of the teeth were on the left and 28.6% (8/28) were on the right. Forty percent (40.3%; 29/72) of the dogs had incompletely erupted LMCs at the time of IP placement, which was commonly (62%; 18/29) bilateral.

### Appliance fabrication

**Figure 2:**
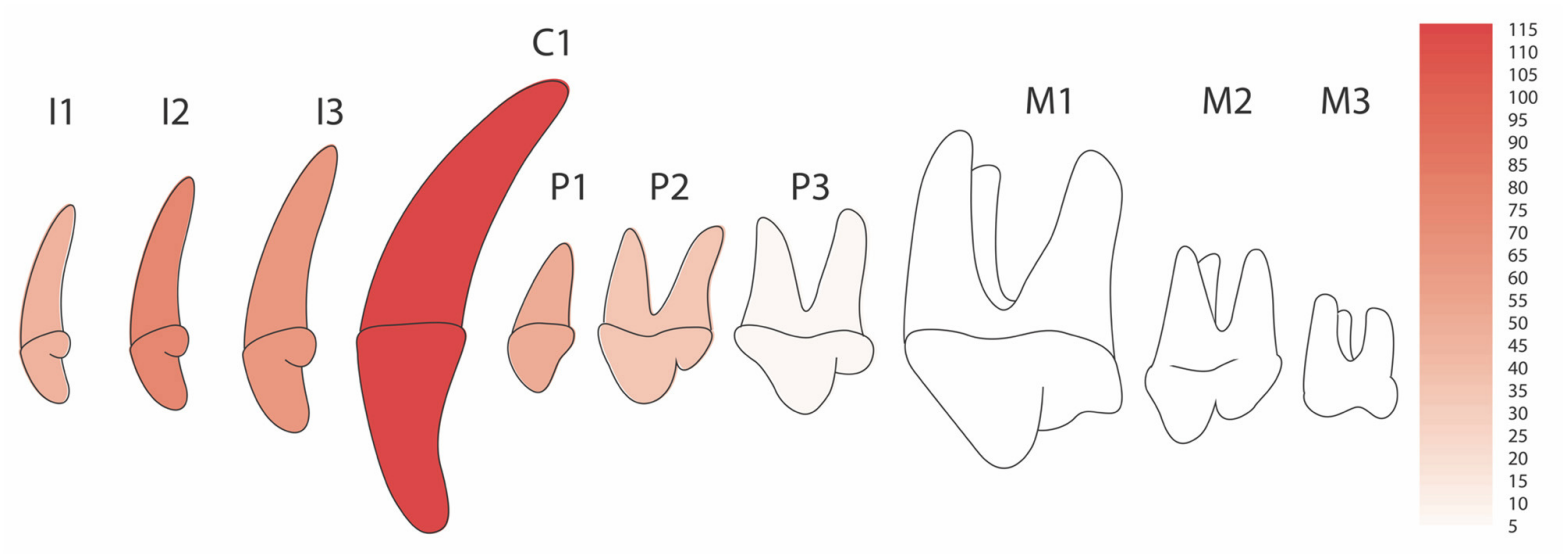
Teeth included in the inclined plane devices. Heat map of the maxillary arcade displaying the frequency of inclusion of each tooth type into the appliance construction. Each tooth represents the inclusion of that tooth whether on the left, the right or both sides. The design of neutral appliances was not included. More I2 were included due to more frequent I3 extractions.

Movement into the interdental space between the maxillary third incisor and canine was more common (86.4%; 102/118) than movement distal to the maxillary canine tooth. There were 19 tooth combinations incorporated into the IP constructs (Figure 2). The median (range) number of teeth included in the IP appliance was 4 (1–10). A midline connection was fabricated as part of treatment in 21.2% (25/118) of cases. Even in cases with midline connections, the left and right sides were analyzed separately. In cases with unilateral disease, a neutral appliance was placed on the contralateral maxilla in 96.6% (28/29) of appliances. Data from the neutral side were not included in the study. In the remaining case, due to the complexity of the class 4 malocclusion present, a neutral IP was not placed.

**Figure 3:**
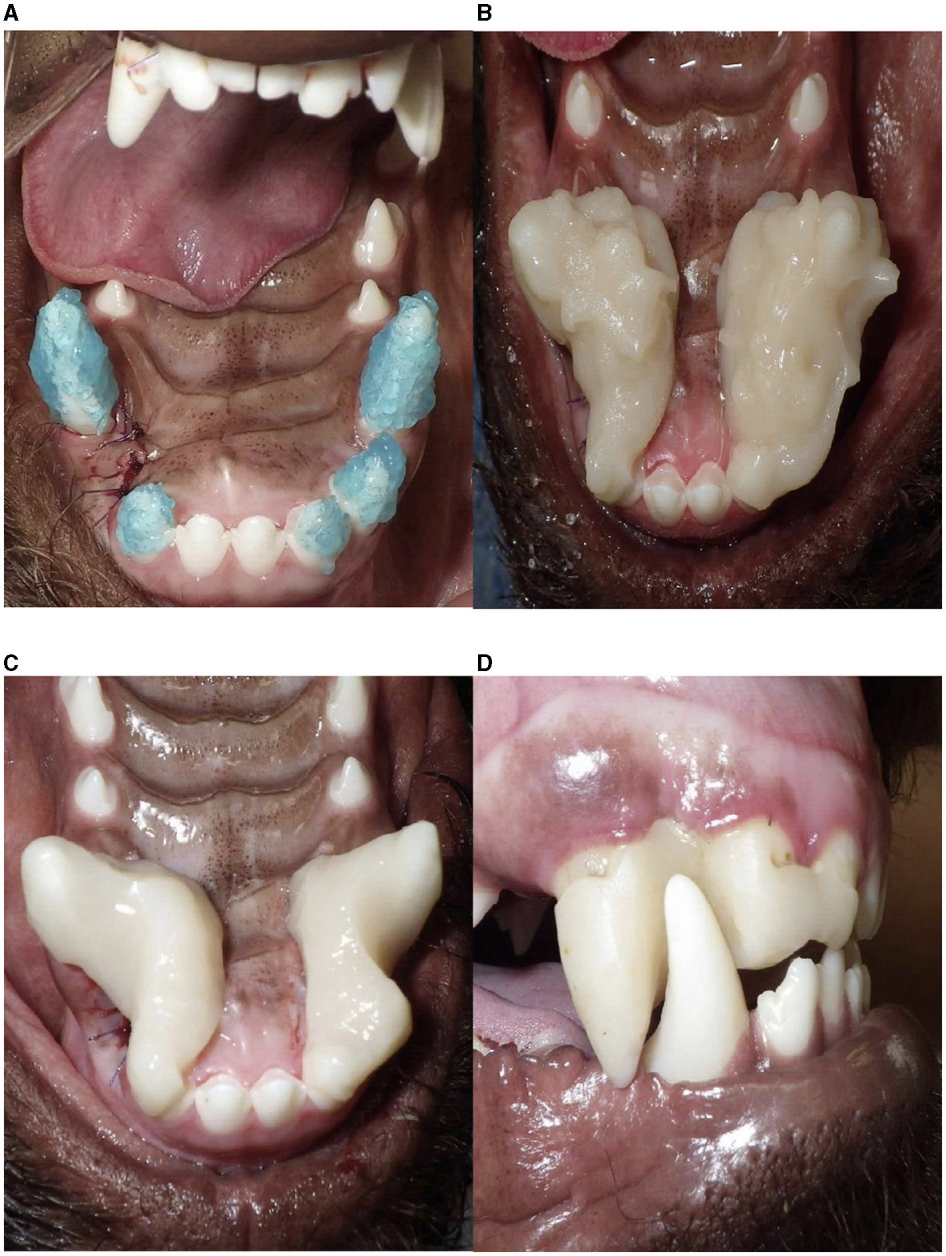
Fabrication and application of an inclined plane. **(A)** Application of acid etch on the teeth to be included into the appliance. Note the extraction site of the left maxillary third incisor tooth, which resulted in varied IP design between left and right. Pending the appliance design, bonding agent may or may not be placed at this time. **(B)** Bulk application of acrylic, **(C)** inclined plane after shaping, **(D)** an appliance placed in a neutral position on the right mandibular canine tooth (no force is placed on the mandibular canine tooth with mouth closure).

Acid etching was performed on the included maxillary teeth prior to IP placement in 95.7% (113/118) of appliances. A bonding agent was applied to the included maxillary teeth in 51.7% (61/118) of appliances. Information on the use of acid etching/bonding agent was not provided in 15.2% (18/118) of appliances. The IP was fabricated with a chemically cured bis-acryl composite in 86.4% (102/118) of the appliances and a light-cured bis-acryl material in 1.7% (2/118) of the appliances (Figure 3). The remainder did not have the material listed. Post-operative recommendations included daily tooth brushing in 79.2% (57/72) of dogs, a chlorhexidine rinse in 94.4% (68/72) of dogs, and soft diet in 75.0% (54/72) of dogs.

### Treatment outcome

Complete resolution occurred in 71.2% (84/118) of teeth. Functional resolution occurred in 25.4% (30/118) of teeth. Incomplete resolution occurred in 3.4% (4/118) of teeth. There were 12 dogs that had variable outcomes between the treated LMC teeth.

No evaluated risk factors, including the age of the patient at the time of IP application, the number of teeth included in the appliance, the type of malocclusion treated, or the number of days that the appliance was in place, significantly (*P* > 0.05) affected the outcome.

The median (range) time that the appliance was in place was 42 (11–174) days. Older dogs had a longer treatment duration than younger dogs, with an increase of 0.048 days of treatment per additional day in age (*P* < 0.006). There was also a significant (*P* < 0.001) increase in treatment duration between class 2 and class 1 malocclusion. The mean (range) treatment time for class 2 was 55 (16–174) days compared to 30 (11–77) days for class 1. There was no significant difference in treatment duration between other classes.

### Adjuvant treatment

Adjuvant treatments were performed for management of the malocclusion in 45.7% (54/118) of teeth and 47.2% (34/72) of dogs (Table 3). Selective extractions were most common and performed in 64.7% (22/34) of dogs. Specifically, concurrent extraction of the third incisor was most frequent and was performed in 72.7% (16/22) of dogs that underwent extractions. Gingivectomy and odontoplasty were performed in 26.4% (9/34) of dogs. Active force appliances were placed at the same time as the IP in 5.9% (2/34) of dogs. Multiple alternative therapies were often performed on a single dog.

**Table 3:** Additional treatments performed for management of the malocclusion in dogs.

	**Breed**	**MAL**	**X**	**GV**	**ODY**	**AOA**
1	Australian shepherd	2	LmandI3			
2	Cardigan Welsh Corgi	3		RmaxI3-C		
3	Cavalier King Charles Spaniel	2		LmaxI3-C		
4	Collie	2	RmandI2+I3 LmandI3	RmaxI3-C LmaxI3-C	RmaxPM4+M1 RmandPM1-M3 LmaxPM4+M1 LmandPM1-M3	
5	Flat-Coated Retriever	1		LmaxI3-C		
6	German Shepherd Dog	1	LmaxPM1			
7	German Shepherd Dog	2				RmaxC LmaxC
8	German Shepherd Dog	2				RmaxC LmaxC
9	German Shepherd Dog	2	RmandI3 LmandI3		RmandI2 LmandI2	
10	Goldendoodle	3	RmandI3 LmandI3			
11	Great Dane	2	RmaxI3 RmandI2+I3 LmandI3			RmandC LmandC
12	Komondor	2	RmandI3			
13	Labrador Retriever	2		RmaxI3-C LmaxI3-C	RmandI1-I3 LmandI1-I3	
14	Labrador Retriever	2	RmandI3 LmandI3			
15	Labrador Retriever	2	RmandI3 LmandI3			
16	Labrador Retriever	2		RmaxI3-C LmaxI3-C	RmaxI2+I3 LmaxI2+I3	
18	Labradoodle	4	RmaxI3 LmaxI3 LmandI3			
17	Labrador Retriever	2	RmandI3 LmandI3		LmaxI2	
19	Labrador Retriever	4			RmaxC RmandC	
20	Labrador Retriever	2	RmandI3 LmandI3			
21	Maltipoo	1	LmaxI3			
22	Miniature Poodle	1	RmaxI1 LmaxI3			
23	Newfoundland	2	LmaxPM1	LmandI3-C	RmandI1-I3 LmandI1-I3	
24	Old English Sheepdog	2				RmaxC LmaxC
25	Pomeranian	3	RmaxI3 LmaxI3		RmaxI1+I2 LmaxI1+I2	
26	Samoyed	2	RmandI3 LmandI3			
27	Soft-Coated Wheaten Terrier	3	RmaxI2+I3 LmaxI2+I3 LmandI3			
28	Spinone Italiano	1		LmaxI3-C		
29	Staffordshire Terrier Mix	1				RmaxC
30	Standard Poodle	1		LmaxI3-C		
31	Standard Poodle	1	LmaxI3 LmandI3			
32	Standard Poodle	1	RmaxI3			
33	Toy Manchester Terrier	1	LmaxI3			
34	Toy Poodle	3	RmaxI3 RmandI3 LmandI3		RmaxI1+I2 LmaxI1+I2	

The teeth were treated with extraction, active orthodontic appliances, and odontoplasty. Gingivectomy is noted with a (-), indicating that the gingiva between the two listed teeth was treated.

MAL, class of malocclusion; X, extraction; GV, gingivectomy; ODY, odontoplasty; AOA, active orthodontic appliance; L, left; R, right; Max, maxillary; Mand, mandibular; I, incisor; C, canine; PM, premolar; M, molar.

The effect of adjuvant therapy on orthodontic success was not statistically evaluated. Of interest, 66.7% (2/3) of maxillary canine teeth treated with an active force appliance applied in conjunction with an IP for orthodontic treatment of the ipsilateral LMC were successful in creating sufficient space between the maxillary third incisor and the maxillary canine tooth for the LMC. The third appliance was dislodged, resulting in the failure of the IP to tip the associated mandibular canine tooth labially but with the successful distal movement of the associated maxillary tooth.

### Peri-treatment complications

While the appliance was in place, 31.4% (37/118) of appliances required at least one appliance adjustment or replacement. Specifically, 81.1% (30/37) of these appliances required one adjustment, and 18.9% (7/37) required two adjustments. Of the 44 total anesthetized adjustments made, the reasons for adjustment included dislodgement (*n* = 8), no longer facilitating tipping movement (*n* = 33), iatrogenic movement to the maxillary canine tooth (*n* = 1), or not listed (*n* = 1).

### Removal and placement of retention devices

**Figure 4:**
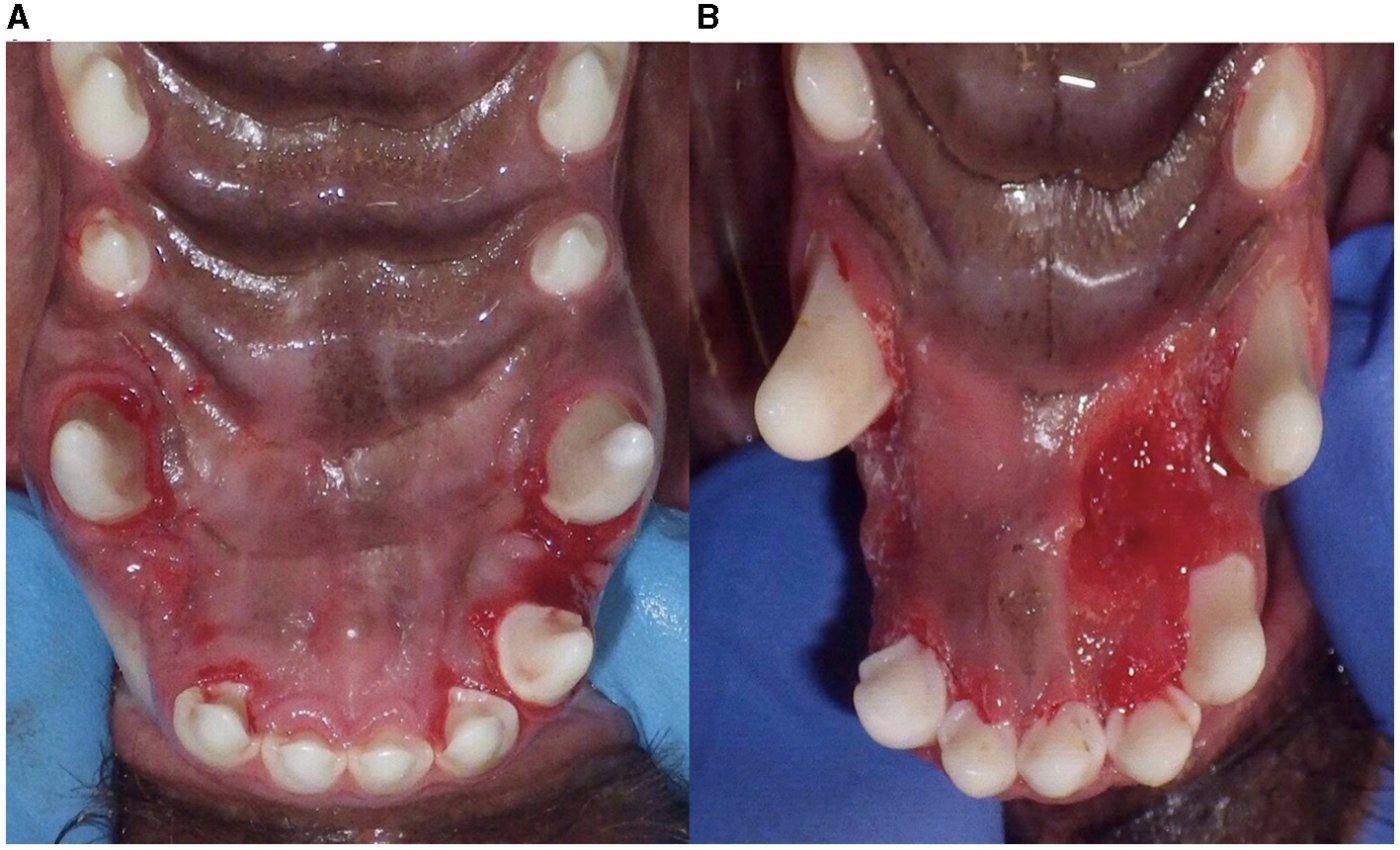
Soft tissue inflammation after removal of the IP device. Gingivitis **(A)** and palatitis in two cases **(B)** after removal of the inclined plane.

Complications occurred during IP removal in 16.9% (20/118) of appliances and 19.4% (14/72) of dogs. A 3-mm gingival laceration occurred in one dog, which healed with no complications following suturing. Tooth staining occurred in the maxillary teeth where the appliance was located in six cases. Enamel fracture occurred in 13 teeth (four maxillary third incisors, six maxillary canine teeth, one mandibular canine tooth, and two maxillary premolar teeth), and enamel-dentin fracture occurred in two maxillary incisors and 1 mandibular incisor at the removal of the appliance. Gingivitis at the appliance margin was seen in 55.5% (40/72) of dogs, and palatitis deep to the appliance was seen in 16.7% (12/72) of dogs (Figure 4). These lesions were completely resolved after the removal of the IP in all cases. Radiographs were performed at appliance removal for 96.6% (114/118) of LMC teeth. No endodontic disease or tooth resorption was noted. Long-term radiographic follow-up was not collected in this study.

Of the 114 teeth with complete or functional resolution, 51.7% (59/114) had a retainer placed at appliance removal. A retainer was recommended when the position of the LMC tooth could not be stabilized by the maxillary gingiva on mouth closure in 69.5% (41/59) of those teeth. Of the teeth with a lack of a natural retainer, 49% (20/41) of the teeth were infraerupted, 27% (11/41) had an associated open bite, and 25% (10/41) were unspecified. In the remaining teeth, 1.7% (1/59) had retention crown extensions placed due to atraumatic gingival contact. The remainder did not have a specific diagnosis.

The retainers used include crown extensions in 44.0% (26/59) of teeth, ball therapy in 35.6% (21/59), and a fixed bis-acryl/wire device placed between the mandibular canine teeth in 20.3% of teeth (12/59). Three teeth that had retention crown extensions placed had secondary tooth fractures, two-thirds with pulp exposure.

Recheck information was available in 80.5% (95/118) of teeth and in 80.6% (58/72) of dogs. The median (range) time to recheck following IP removal was 31 (2–1,557) days. Follow-up was performed in person on 74.6% (88/118) of teeth and 76.4% (55/72) of dogs. Email/phone follow-ups were performed on 5.9% (7/118) of teeth and 6.9% (5/72) of dogs. Of note, 19% (19.5%; 23/118) of teeth and 19.4% (14/72) of dogs were lost to follow-up.

A total of 14% (14.4%; 17/118) of all teeth had rebound movement. If only including dogs that had recheck information available for review, 17.9% (17/95) of teeth had rebound movement. Additional treatment under general anesthesia was recommended in all dogs, yet it was only performed in 76.5% (13/17) of these teeth and in 81.8% (9/11) of dogs (Table 4).

**Table 4:** Treatments performed after the removal of the IP device due to the rebound of the LMCs in 11 patients.

	**Breed**	**MAL**	**Treatment**	**Reason**
1	Australian Shepherd	2	CE	LMC relapse
2	Cairn Terrier	2	IP	LMC relapse
3	Collie	2	IP	LMC relapse
4	German Shepherd	2	Declined treatment	Maxillary and mandibular canine tooth contact on mouth closure
5	German Shepherd Dog	2	GV	LMC relapse
6	Labrador Retriever	2	IP	LMC relapse
7	Miniature Dachshund	2	Declined ODY or CE	LmandC contacting distal LmaxC with gingival recession
8	Newfoundland	2	GV/Ball	LMC relapse
9	Spinone Italiano	2	GV/CE	LMC Relapse
10	Spinone Italiano	1	GV/Ball	LMC relapse
11	Standard Poodle	1	VPT	LMC relapse

MAL, class of malocclusion; GV, gingivectomy; Ball, ball therapy; IP, inclined plane; ODY, odontoplasty; CE, crown extension.

### Success compared to crown extensions

The relative success rates, distribution of malocclusion, and severity of linguoversion before treatment with either crown extensions (2) or IP are shown in Table 5. The complete and functional success rate is very similar with both treatment options, although rebound is higher with IP.

**Table 5:** Comparison of treatment groups and outcomes for LMCs when evaluating the success of crown extension (CE) (2) and inclined plane (IP) appliances.

	**IP**	**CE**
Number of treated dogs	72	72
**Class of malocclusion in dog (%)**		
MAL1	41.7	73.6
MAL2	47.2	19.5
MAL3	6.9	6.9
MAL4	4.2	0
**Severity (%)**		
Mild	7.6	34.7
Moderate	33.9	44.4
Severe	58.5	20.8
**Outcome (%)**		
Complete resolution	71.2	77.8
Functional	25.4	20.8
Incomplete	3.4	1.4
Tooth rebound (%)	14.4	1.4

The severity of the malocclusion and the outcome were evaluated by the tooth in the IP study due to variation between the teeth in the same dog. For patients treated with CE, the severity and outcome were assessed for each dog rather than each tooth. The outcome for the IP was determined at the time of IP removal. In some cases, the CE was assessed after a period of time after appliance removal and after a retention period. Rebound was evaluated at a recheck appointment after the removal of the appliance in both cases.

## Discussion

Complete correction or functional outcome was seen in 96.6% of LMC teeth treated with an IP. This is the first study to document that directly applied IP is a highly successful treatment option for a variety of malocclusions that may result in LMCs.

The median treatment time for resolution was ~6 weeks, with some teeth having resolution in as quickly as 11 days. The primary contributors to the speed of orthodontic movement were the class of malocclusion and the age of the patient. Dogs with class 2 malocclusion had a significantly longer treatment duration with a mean of 55 days compared to 30 days for class 1. In Beagle dogs, the maximum rate of orthodontic movement has been documented at 2.5 mm per month (17). Mandibular distocclusion (class 2 malocclusion) increases the distance required to move the tooth into the anatomical space between the maxillary third incisor and canine. Thus, placement of the LMC distal to the maxillary canine tooth may be a more pragmatic treatment option to decrease treatment time. An alternative option is to orthodontically move the maxillary canine tooth distally, making more space in the interdental space between the maxillary canine and the third incisor. In this study, an active force orthodontic appliance to move the maxillary canine tooth distally was placed in 5.9% (7/118) of the teeth and 5.6% (4/72) of dogs. Almost half (42.8%, 3/7 teeth) of these were treated concurrently with the IP, while the remaining were treated prior to IP placement.

The maxilla and the mandible respond differently to orthodontic forces, with similar forces resulting in almost double the spatial movement in the maxilla compared to the mandible (18). This difference has been explained by the thinner maxillary cortices, differences in the orientation of the trabecular bone, and increased rate of bony turnover seen in the maxilla (19). This makes concurrent treatment of the maxillary and mandibular canine teeth reasonable as the maxillary canine tooth is likely to move distally at an increased rate compared to the mandibular canine tooth, creating space for the LMC. To accomplish concurrent treatment, the IP must be applied to the maxillary canine tooth only. Concurrent treatment with an IP and an active orthodontic device was successful in treating these malocclusions simultaneously in one case report (14). In this study, 66.7% (2/3) of the teeth treated concurrently with an active force appliance were successful. The third appliance was dislodged, resulting in the failure of the IP. Future studies should focus on evaluating the success rate of this combined treatment in a larger cohort.

The median age for patients undergoing IP treatment was 223 days. This demographic is consistent with dogs treated in previous orthodontic studies (2, 13). Unsurprisingly, older dogs had an increase in treatment time of 0.048 days per day of age increase. Younger dogs respond faster to orthodontic forces due to active bone remodeling, decreased density of cortical bone, and the contribution of the eruptive tooth force in moving the tooth (20). Furthermore, there is a delayed cellular response (increase in prostaglandin E2, Interleukin-1 receptor, RANKL, and osteoprotegerin) to orthodontic forces in older patients. Multiple studies in both humans and animals have shown an up to two-fold difference in the speed of orthodontic displacement in younger patients (18, 20–22).

Interestingly, varied appliance configurations (number of teeth, movement mesial or distal to the canine tooth, presence of a midline connection, materials used, addition of an active force appliance) all appeared to result in a good outcome within a reasonable treatment duration. Yet, only the number of teeth in the appliance was evaluated statistically. Nineteen tooth combinations were used in the appliances with a mean of four teeth incorporated. The design typically incorporates a combination of maxillary incisor, canine, and premolar teeth. The final design of the appliance relies on a number of factors. Maxillary tooth extraction was also performed in 18% (13/72) of dogs at the time of appliance placement. This limits the teeth available for fixation of the appliance. The concurrent use of an active orthodontic device, particularly in the treatment of mesioversion of the maxillary canine, will also limit the application of the IP appliance to the maxillary canine tooth only.

A connection between the left and right appliances has also been described, although a concern that fixation may affect maxillary growth has been raised (3, 11, 15). The primary concern is that the appliance may inhibit the widening of the maxilla at the intermaxillary suture and lengthen at the incisivomaxillary suture (23). Yet, multiple forces affect the growth and stability of the maxilla. Genetics, soft tissue forces of the tongue, lips, and muscles, dental interlock, and occlusal forces continuously act on the maxilla. Furthermore, the intermaxillary suture has been documented to be stable in dogs at 25 weeks of age, so by the time of orthodontic placement, disruption is unlikely (23). Of note, this period may be longer in larger-breed dogs that continue to have skeletal growth until 18 months of age or longer (24). Using a telescoping connection at midline or creating independent left and right devices will allow for continued growth at this suture (25). In this study, 19.4% (14/72) of the dogs had a midline connection. A clinically relevant discrepancy in maxillary width or length was not appreciated. The IP design did not affect function or success, and it appears that a variety of tooth combinations and design features can be successful in managing these cases.

Both the complete and functional success rates documented with IP were similar to those historically reported for crown extensions (2). Yet, the IP-treated cases had a larger proportion of both more complex skeletal malocclusions and more severe LMC displacement. IP may be a better orthodontic treatment option for severe cases. However, this difference in severity may be related to selection bias; thus, further research is required to confirm this assumption.

The primary disadvantage of orthodontic appliances as compared to other treatment modalities is that they require a minimum of two anesthetic events for placement and removal. Additional anesthetic events may also be required. In this cohort, 31.4% of the dogs required at least one anesthetized appliance adjustment. Appliance adjustments were most commonly needed when an appliance ceased to tip the LMC appropriately. Patients who are heavy chewers may need more frequent adjustments. In these patients, considering indirect methods that allow for sturdier materials, such as cast metal, may be a better option than the bis-acryl resin used in this study. However, cast metal and other indirect methods require a minimum of three anesthetic procedures. Alternatively, crown reduction with endodontic therapy or surgical extraction may be more predictable.

The other main disadvantages of orthodontic appliances are the potential for soft tissue inflammation, trauma secondary to appliance removal, iatrogenic movement of other teeth, secondary tooth resorption, and rebound movement. Severe complications associated with direct IP were not noted within this cohort, while minor self-limiting complications occurred frequently. Soft tissue inflammation was the most common complication noted, with gingivitis and palatitis noted in 55.5% and 16.7% of cases, respectively. Proper appliance design to maintain a space of 1–2 mm from the gingival margin to help prevent this, as well as daily homecare with rinsing solution and tooth brushing, can minimize this complication. In this study, soft tissue inflammation resolved without intervention by the initial recheck in all cases. Furthermore, upon the removal of the appliances, it was noted that enamel or enamel-dentin fractures occurred in 12.7% (15/118) of appliances. Acid-etching and application of a bonded sealant prior to acrylic application may help prevent dislodgement of the device but also make removal more challenging. Keeping the cusps of the included teeth uncovered during acrylic application, careful scoring of the acrylic material at an edentulous area, such as the interdental space between the maxillary third incisor and canine teeth, using a bur, and careful removal of the remaining acrylic with a variety of sharp instruments are recommended. Severe fractures requiring endodontic treatment did not occur during appliance removal for any dog in this study.

With occlusal contact by the LMC, the IP appliance can inadvertently create force in an apical direction that may result in intrusion of the LMC because intrusion requires less force than tipping (26). This type of force may prevent or slow the continued eruption of the LMC. In this study, 41.5% (40/118) of the treated teeth were partially erupted at the time of IP placement. This could be due to the age of the animal, occlusal contact with the palate that creates a physical obstruction to eruption, or secondary tooth retention of these teeth. Due to the lack of thorough follow-up, it is unknown if the teeth that were infraerupted ever fully erupted or if this was precluded by constant occlusal force from the IP. This is an area that should be further explored in future research as a potential severe long-term complication of IP placement.

Palatal displacement of a left maxillary canine tooth was noted in one tooth. This particular appliance included the canine tooth, first premolar, and second premolar tooth with the aim of moving the LMC distally to the maxillary canine. In theory, this should have been avoided by appliance design. The appliance is meant to form an anchorage unit to resist the force of the LMC on mouth closure (27). The ideal combined root surface area of the anchor teeth is twice that of the teeth being moved. Periodontal compromise of the maxillary canine tooth with the higher propensity of the maxillary teeth for orthodontic movement may have allowed for the inadvertent movement despite creating an anchorage point. In this case, consideration may have been given to adding a midline connection and creating more palatal coverage to increase the anchorage resistance (28). Palatal displacement or other inadvertent movement of anchor teeth has not been reported in previous case reports and is likely rare.

Excessive/rapid orthodontic force can result in ischemic necrosis of the periodontal ligament, resulting in tooth resorption, and can disrupt pulpal blood flow, resulting in non-vitality (26). The IP is a passive orthodontic device that relies on intermittent patient-controlled force, so excess force on the periodontal ligament and resultant side effects are unlikely. Furthermore, the human literature largely supports the idea that changes to pulpal blood flow with orthodontic therapy are transient and insufficient to result in non-vitality (29, 30). Radiographs of both the orthodontically treated teeth as well as those that provided anchorage are recommended. No overt evidence of tooth resorption or non-vitality was noted radiographically. However, normal radiographs do not eliminate the presence of disease as changes may not be apparent until 30%−40% of mineral content is lost, and apical periodontitis may not be visible for at least 30 days (31). Furthermore, in human studies, histologic studies showed a 90% occurrence of root resorption that was not reflected in radiographic findings (32). A radiographic recheck in 3–6 months following IP treatment is recommended.

Lastly, rebound movement of orthodontically treated teeth is possible. Following IP treatment, 14.4% of treated teeth required additional treatment due to rebound tooth movement. Comparatively, crown extension treatment was associated with only 1.4% rebound (2). The increased severity/displacement of the teeth treated in the IP study, as well as the fact that the outcome of the CE was often assessed after a retention period was performed, likely affected this outcome. Due to the retrospective nature of this study, it was unclear if the total time the IP was in place was based only on the time required for the movement of the teeth or if it also represented a retention period based on surgeon preference. In general, most dentists elect to remove the IP device when the teeth have moved into place as the appliance can be irritating to tissues; thus, the time the appliance was in place is likely analogous to the time of tooth movement. Yet, this is speculation only. In this study, 81.8% (9/11) of the dogs that were retreated had a class 2 malocclusion.

Both extrinsic and intrinsic forces can determine if rebound tooth movement is likely. The primary intrinsic forces of concern are the residual forces in the tissues of the periodontium, which remain until the tissues have finished remodeling (33, 34). It can take some of the fibers up to 1 year to remodel, allowing rebound movement to occur (35). Extrinsic forces, such as ongoing skeletal growth, soft tissue pressures, and tooth interdigitation, can also impact the likelihood of rebound movement (36). Deep tooth interdigitation, as is typical in the occlusion of canine teeth in dogs, is likely to maintain stability after treatment and act as a natural retainer (20, 34, 37). The veterinary literature recommends a retention duration of half the total treatment time and can be performed with the same device used for orthodontic movement (20). There are no clear recommendations in human orthodontics for a retention period, and relapse remains unpredictable (33, 34). These authors recommend a retainer be placed after IP removal if there is not a robust natural canine interlock. Yet, the addition of a retainer is not without potential complications. Due to the increased height of the tooth with crown extensions, there is an increased risk of tooth fracture (38). Within this study, nearly 8% (2/26) of teeth sustained a fracture secondary to retentive crown extensions, which required endodontic treatment. Furthermore, future anesthetic events will be required to remove the retention device after a sufficient retention period. The IP may be considered for short-term retention to reduce additional anesthetic events. However, given the frequency of soft tissue inflammation secondary to the appliance, replacement of the device for long-term retention may be beneficial for patient comfort.

The retrospective study design allowed for a broader evaluation of this procedure. However, this study type presents distinct limitations. A lack of standardization of the procedure and clinical decision-making between cases may have influenced outcomes. Pre-treatment and post-treatment photographs were not available for all cases. Furthermore, ~25% of the cases had no follow-up after the removal of the device, and the follow-up that was available was inconsistent and, in some cases, given by the owner. This may have resulted in an underestimation of changes over time. Eleven of the dogs evaluated had some degree of LMC rebound. Many cases had retention plans after treatment. Further information on the motivation to use retention, the duration of, and the outcome of this treatment may have provided updated recommendations for this aspect of orthodontic treatment. Despite these limitations, this is the largest study to date that evaluates the success rate of direct IP in dogs. It can be concluded that this is a successful and predictable procedure, with only minimal complications noted. Yet, owner commitment to the process is paramount as additional treatments, additional anesthetic events, and ongoing retention may be needed.

## Data availability statement

The raw data supporting the conclusions of this article will be made available by the authors, without undue reservation.

## Author contributions

LT: conception and design of the study, data collection, interpretation of the data, and drafted the manuscript. SG: interpretation of the data and revision of the manuscript. LL performed statistical analysis. All authors contributed to the article and approved the submitted version.

## Footnotes

^1^American Veterinary Dental College. *AVDC Nomencl Occlusal Abnorm* https://avdc.org/avdc-nomenclature/ (accessed April 28, 2023).
